# Promoting student confidence in the OSCE process

**DOI:** 10.1080/10872981.2017.1324719

**Published:** 2017-05-19

**Authors:** Abdulla Bedir, Ahamodur Choudhury, Sheikh Naheed Chowdhury

**Affiliations:** ^a^ King’s College London, Guys Kings St Thomas’ School of Medicine, London, UK

We would like to begin by thanking Reid et al. for their captivating paper ‘Taking OSCE Examiner Training On The Road: Reaching The Masses​​’ [1]. As senior year medical students we read this article with keen interest and share the belief that further development of systematic examiner training will result in fairer, more objective clinical assessments. Moreover, we believe that transparency alongside this also contributes to the reliability of the examination process, and is important for student confidence in OSCEs (objective structured clinical examinations​​) as a means for assessing their competency and their personal development as a future doctor.

So what particularly stood out to us was the high turnout of medical students at the grand round training described in the study. Although reasons for this may have been multifactorial, it was unsurprising, and we believe it highlighted the high degree of uncertainty that exists amongst medical students concerning the OSCE administrative process. Indeed, it is our experience that details surrounding the mechanics of OSCE administration are largely ambiguous. What makes this of particular concern, however, is that studies have shown that anxiety and uncertainty regarding test methodology can be detrimental to test reliability [2]. It would therefore be interesting to investigate the effect that medical student exposure to the grand rounds had on student perception, and while negative perceptions amongst medical students remain despite historical efforts to reduce bias [3], we believe that increased transparency in how examiners are recruited and trained as well as how stations are processed is likely to promote confidence in the objectivity of the format.

In addition, and as discussed by the authors, more has to be done to improve OSCE standardisation and uphold objectivity with regards to the marking of student performances at examination. How alternative approaches to clinical skills and OSCE stations are accounted for by examiners, who themselves have differing expectations, is another big concern. A recent study of an established OSCE testing centre identified several common examiner factors that continue to limit OSCE reliability despite intensive training and experience [4]. Yet this issue was not addressed as a factor in the study, and the effects of different station types and staff grading could also have been explored.

Aim should be taken at the impact that these variables have on the reliability of clinical OSCEs, as they preclude the formation of a clear benchmark for students. In the context of this study, one solution could involve expanding examiner acceptance for alternative techniques by demonstrating multiple simulated OSCE videos showing all acceptable approaches as well as performances across the spectrum from clear pass to fail as the study had trailed. However, where this may prove challenging to implement, central core teaching is a notable alternative with many students reporting a lack of clinical skills training at external sites [3].

Ultimately, standardisation of OSCEs is critical to the formation of a strong benchmark, for which thorough training of examiners is integral [5]. And it is transparency that enables students to refer to this benchmark and assimilate feedback which would otherwise be uncertain and un-actionable. Both aspects are likely to be strong determinants of student confidence in the OSCE process, and it is interesting to acknowledge the potential that recent adoption of video technology by many medical schools has in enabling these to be addressed [6]. In these efforts, the integrity of OSCE examinations must be strongly upheld. As such, transparency to promote confidence should extend only as far as to demonstrate the objectivity and standardised nature of OSCEs that is otherwise assumed of written examinations. It should not extend to detailing marking and assessment at a granular level that would undermine the fostering of a deep understanding of clinical skills amongst medical students, which should always be the focus.

Given that student confidence in the OSCE process contributes significantly to not only overall student satisfaction but likely impacts OSCE performance and the acquisition and retention of clinical skills, we believe that efforts should be exhausted to promote student confidence and tackle the key issues that undermine it. That includes efforts to enhance standardisation and promote transparency. As the authors have rightfully emphasised, there is need for further objective research.
